# A pragmatic cluster randomized trial evaluating the impact of a community pharmacy intervention on statin adherence: rationale and design of the Community Pharmacy Assisting in Total Cardiovascular Health (CPATCH) study

**DOI:** 10.1186/1745-6215-11-76

**Published:** 2010-07-08

**Authors:** Charity D Evans, Dean T Eurich, Jeff G Taylor, Alfred J Remillard, Yvonne M Shevchuk, David F Blackburn

**Affiliations:** 1 College of Pharmacy and Nutrition, University of Saskatchewan, Saskatoon, Canada; 2 School of Public Health, University of Alberta, Edmonton, Canada

## Abstract

**Background:**

Traditional randomized controlled trials are considered the gold standard for evaluating the efficacy of a treatment. However, in adherence research, limitations to this study design exist, especially when evaluating real-world applicability of an intervention. Although adherence interventions by community pharmacists have been tested, problems with internal and external validity have limited the usefulness of these studies, and further well-designed and well-conducted research is needed. We aimed to determine the real-world effectiveness of a community pharmacy adherence intervention using a robust study design. This novel design integrates cluster randomization and an outcome evaluation of medication adherence using a population-based administrative data source in the province of Saskatchewan, Canada.

**Methods/Design:**

Community pharmacies from across the province of Saskatchewan, Canada were randomized to deliver an adherence intervention to their patients or usual care. Intervention pharmacies were trained to employ a practical adherence strategy targeted at new users of statin medications. While randomization and implementation of the intervention occurred at the community pharmacy level, the outcome analysis will occur at the level of the individual subjects. The primary outcome is the mean statin adherence among all eligible new users of statin medications. Secondary outcomes include the proportion of new statin users who exhibit adherence ≥80%, and persistence with statin use.

**Discussion:**

This novel study design was developed to combine the rigor of a randomized trial with a pragmatic approach to implementing and capturing the results in a real-world fashion. We believe this approach can serve as an example for future study designs evaluating practice-based adherence interventions.

**Trial Registration:**

ClinicalTrials.gov no. NCT00971412.

## Introduction

Randomized controlled trials (RCT) are considered the gold standard design for the evaluation of treatment efficacy [1], and are increasingly being used for evaluating interventions aimed at improving medication adherence [2]. However, in adherence research, limitations to this study design exist. First, traditional RCTs require that all study participants are informed and consenting volunteers. This requirement likely pre-selects individuals who are at a lower risk for non-adherence, particularly for RCTs of behavioural interventions. Indeed, patients enrolling in clinical trials may be systematically different than those declining to participate[2,3]. In addition, when traditional RCT designs are used, the same health care professional is often forced to provide the adherence intervention and 'usual care' concurrently [4,5]. Clearly, contamination is unavoidable when the same health practitioner is providing the control and experimental conditions simultaneously [3].

Because of their accessibility and frequent interactions with patients, community pharmacists are considered ideally situated to deliver interventions aimed at improving medication adherence [3]. However, adherence interventions by community pharmacists have not consistently shown benefits [3,6]. One of the reasons may be that these studies are often plagued with methodological problems. Many studies do not report a power calculation, do not utilize proper randomization techniques or allocation concealment, and most do not provide adequate details of the intervention when reporting the results [7]. Furthermore, generalizability of successful interventions is often low because most health care professionals do not have the time or resources to implement complex study protocols [8-11]. As a result, little is known about the extent to which community pharmacists can influence adherence in real world settings.

We describe the design of a study aimed at evaluating the real-world effectiveness of an adherence intervention provided by community pharmacies; the Community Pharmacy Assisting in Total Cardiovascular Health (CPATCH) study. This prospective study employs a novel design that integrates cluster randomization with an evaluation of *all *at-risk patients using an administrative database. This design presented herein was developed to strengthen the external validity of research studies that examine adherence interventions while maintaining the high standards required for prospective clinical trials. Although the general CPATCH strategy is intended to be employable with all cardiovascular medications, this study is focused on the prevention of non-adherence among new users of statin medications. Statin medications were chosen for this initial study, as it is well known that 40% to 50% of all new statin users become non-adherent within the first year [12,13], and a sizeable proportion discontinue the medication after only one dispensation [14].

## Methods

### Recruitment of Community Pharmacies

In the Fall 2009, all community pharmacies across the province of Saskatchewan (n = 357) were invited to enrol in the CPATCH study. Advertisements were placed in bulletins and newsletters distributed by provincial pharmacy organizations. Also, individual letters were mailed to all pharmacy owners/managers in the province and the study investigators advertised in person at various local pharmacy conferences and events.

Community pharmacies interested in enrolling were directed to the study coordinating centre at the University of Saskatchewan to determine their eligibility. Pharmacies were eligible for enrollment if: i) they filled at least 85 total statin prescriptions during a consecutive 6 week period; ii) the owner/manager indicated that this adherence intervention would be given priority over all other study initiatives offered by the pharmacy staff; and iii) all members of the pharmacy (dispensary) staff provided informed consent.

### Randomization

The CPATCH study employed a clustered randomized trial design whereby community (retail) pharmacies were randomized into one of two groups: an intervention group that received training to provide the CPATCH adherence strategy to their patients; and a usual care group that did not receive any specialized adherence training and will provide their patients with standard care.

A computer generated randomization list, in permuted blocks of 6, was created by an employee at the Drug Information centre at the University of Saskatchewan who is not an investigator in this study. Each randomly allocated group assignment (intervention or usual care) was individually sealed in sequential, opaque envelopes and kept in the office of the Drug Information Centre. Given the nature of the intervention, it was impossible for the study investigators and pharmacy researchers (i.e. participating pharmacies) to be blinded to the group assignments; however, all study researchers were blinded to the sequence generation, and all allocations were concealed until the study group was assigned. Furthermore, randomization was carried out in multiples of 6 (i.e., six pharmacies were randomized at a time) to ensure that the investigators had no opportunity to anticipate which group a pharmacy would be allocated to.

### The Intervention

Pharmacies (i.e. pharmacists and pharmacy technicians) randomized to the intervention group received training on how to implement the CPATCH support program within their store; the control pharmacies did not receive any training, and were intentionally not given any indication of the focus of the study intervention (adherence of new statin users). Training on the CPATCH strategy was provided through a 2.5 hour workshop delivered in a setting outside the dispensary. Briefly, the training program introduced a simple and coordinated strategy to consistently identify and support patients at high risk for statin non-adherence. The criteria used to identify individuals at high-risk for non-adherence were based on epidemiologic observations of statin use. Specifically, individuals receiving their first dispensation for a statin medication have a 40%-50% of becoming non-adherent in the first year of therapy [12,13]. Further, many patients will discontinue after only one dispensation [14]. These dispensation patterns were used to provide objective evidence about the risk for non-adherence associated with new prescriptions for these medications. Also, these data were used to justify our hypothesis that initial statin dispensation encounters are critical to the prevention of non-adherence.

The CPATCH strategy was designed specifically to be implemented into real-world community pharmacy practice without the need for additional resources or significant changes to existing workflow procedures. As a result, the program is flexible to each specific pharmacy. The consistent elements of the overall CPATCH training strategy are: i) routine identification of new users (those in their first year of therapy) of statin medications as they present for each dispensation; ii) consistent assessment for barriers to non-adherence at every dispensation for at-risk patients (new users); iii) a paradigm shift from traditional counselling to an approach that focuses on patient preparation and reassurance; preparation for the negative messages about statins they will likely hear in the coming months and reassurance that this medication is backed by objective evidence, and iv) a commitment by pharmacy staff to actively respond to identified adherence barriers such as cost, intolerance, lack of knowledge, or beliefs about the medication. The focus of this strategy is to lower the incidence of non-adherence rather than remedy those patients with established non-adherence.

### Subjects

Intervention pharmacies will identify patients within the first year of statin therapy through dispensation records and face-to-face encounters. Although intervention pharmacies will target all statin users within their first year of therapy, data will only be collected on those subjects who: i) receive a new statin prescription from a study pharmacy (intervention or control) during the observation period; ii) have no statin fills recorded during the year prior to the index prescription (first fill for a statin medication during the study observation period); and iii) have been continuously enrolled as a Saskatchewan Drug Plan beneficiary for at least 365 days prior to the index prescription. These study subjects will be identified independently by personnel from the Saskatchewan Ministry of Health and all outcome data will be collected at the administrative database level (see "Follow-up").

The observation period for each pharmacy will begin two weeks after their training session. This two week lag period will be used to allow each staff to implement the strategy and resolve any issues with the help of the CPATCH investigators. To ensure consistency between groups, usual care pharmacies were assigned a start date that matched the date of an intervention pharmacy falling most closely in the order of randomization. As a result, in each permuted block of six randomized pharmacies, three pairs (one control and one intervention) of stores will share the same start date.

### Follow-up

All patient information and outcome data will be collected from administrative databases maintained by the Saskatchewan Ministry of Health (Sask Heath). Sask Health maintains a registry and several databases containing health-services records such as prescription drug data, hospital services data, and physician services data (Table 1). Health services information captured by distinct databases can be linked at the patient level through a unique identification number for each individual. These databases have served as the basis for many observational studies because they capture data on the vast majority of Saskatchewan residents [15-18]. For this study, the databases will be used to capture health services utilization (prescriptions, physician visits and hospitalizations) including diagnoses on all eligible subjects.

**Table 1:** Relevant Data Elements Available from Saskatchewan Health Databases [18]

Database	Available Data
Prescription Drug	Patient information
	Drug information
	Prescriber information
	Dispensing pharmacy information
	Cost information

Hospital Services	Patient information
	Diagnostic and procedure information (based on ICD-10-CA^a ^and CCI^b^)
	Admission and discharge dates
	Length of hospital stay

Physician Services	Patient information
	Physician information
	Diagnostic information (3 digit ICD-9^c ^code)
	Billing information

Insurance registry	Beneficiary information (effective date, end date, sex, age, residence)

a. International Statistical Classification of Disease and Related Health Problems, Tenth Revision, Canada [35]

b. Canadian Classification of Health Interventions [36]

c. International Classification of Disease, Ninth Revision

All Saskatchewan residents are eligible for provincial health insurance coverage, except inmates of federal penitentiaries and members of the Royal Canadian Mounted Police and Canadian Forces (less than 1% of the Saskatchewan population). All health insurance beneficiaries are eligible for Saskatchewan Drug Plan benefits except those who receive these benefits from another government agency, primarily registered Indians (about 9% of the population). Therefore about 10% of the Saskatchewan population is ineligible for the study because of no or incomplete capture of health services information.

The researchers will provide Sask Health with the list of participating pharmacies and their specific Saskatchewan Drug Plan identifiers (i.e., provider identifier), and enrolment dates. Sask Health personnel will extract all statin prescriptions dispensed from the participating pharmacies during the 18 months following the pharmacy's enrolment date. The individuals receiving these prescriptions will form the pool of potential new statin-users. The prescription histories of the potential pool members will be reviewed to determine which subjects are new statin-users. Hospital, physician and prescription information for eligible new statin-users will be compiled and de-identified prior to release to the researchers for analysis.

It is estimated that each pharmacy will need six months to accrue their sample size of new statin users, thereby allowing 12 months for follow up. However, because it will not be possible to determine when each pharmacy's sample size has been met, data will be collected on all new-users presenting to each pharmacy throughout the observation period. Subjects will be followed from one year prior to their index prescription until the end of the pharmacy's observation period, death, or loss of beneficiary status, whichever is sooner.

### Outcomes

Although community pharmacies are the unit of randomization, our primary outcome is the mean statin adherence among all eligible new users of statin medications who have been followed up for at least 6 months past their index prescription. This primary outcome will be measured at the individual level. All statin dispensations for each subject will be captured by the prescription drug plan database and mean adherence will be measured using the proportion of days covered (PDC) [19,20], adjusted for any days that a subject may be hospitalized during the observation period. The PDC is calculated by taking the sum of the days' supply for all statin prescription fills during the study period, divided by the number of days of observation. Study participants who have statin dispensations from non-study pharmacies will be captured and accounted for using methods previously published [21]. Secondary outcomes include the proportion of new statin users who exhibit adherence (PDC) ≥80%, and the persistence with statin use among patients with a minimum of 12 months of follow up. Persistence is defined as the number of days from the index prescription to the earliest occurrence of study end date or date of discontinuation (assumed when a refill is not obtained within 102 days of finishing the estimated supply) [22,23]. Only those subjects with a minimum of 6 months of follow-up will be included in the secondary analyses.

### Sample Size

Using data from a previous observational study evaluating one-year statin adherence of new users at 34 different community pharmacies in Saskatchewan [21], we were able to estimate an intracluster (or intraclass) correlation coefficient (ICC) of 0.0143, and determine that mean statin adherence in this population was 0.71 (SD 0.13). Thus, in order to detect a 15% improvement in mean adherence, considered to be clinically important [24,25], 270 subjects would be required in each group to detect a difference of approximately 15% with 80% power at a p < 0.05. The community pharmacies enrolled in CPATCH are expected to accrue a minimum of 25 subjects over a 6 month period. As a result, 22 clusters (pharmacies) are calculated as needed, but 30 will actually be enrolled as the planned statistical analysis requires a minimum of 15 clusters within each group [26].

### Statistical Methods

Baseline characteristics will be compared between groups using both cluster and individual-level variables (Table 2). To determine the effect of the intervention, mean adherence will be measured at one year using generalized estimating equations (GEE) with an exchangeable correlation matrix and robust standard errors, with individuals as the analytical unit [27]. Both univariate and multivariable models will be developed based on individual and pharmacy-level variables considered statistically significant (P < 0.10), or clinically important (e.g. age, sex) (Table 2). All first order interactions will be tested.

**Table 2:** Individual and Pharmacy Level Variables Available for Multivariable Analysis

Variable	Description	Database Source
***Individual Level***		

Sex	Sex of subject	Insurance registry

Age	Age of subject at index date (calculated using date of birth)	Insurance registry

Income security benefits	Patient receives income security benefit (a proxy for socioeconomic status)	Prescription drug

Concurrent medications	Concurrent medications use during one year prior to subject's index date, and for the first 6 months of the study observation period.	Prescription Drug
	Beta blockers	
	Angiotensin converting enzyme inhibitors	
	ASA	
	Oral hypoglycemics	

Prior cardiovascular event	Cardiovascular event(s) in one year prior to subject's index date (Based in ICD-10-CA classifications)	Hospital Services
	Myocardial infarction (I21. - I23.)	
	Stroke (all causes) (I60. - I64.)	
	Transient ischemic attack (G45.0)	
	Unstable angina (I20.0)	
	Other ischemic heart disease (I24. - I25.)	
	Revascularization procedures	
	PTCA (1.IJ.50, 1.IJ.57.GQ, 1.IL.35), CABG (1.IJ.76)	
	Coronary Angiography (3.IP.10)	

Study cardiovascular event	Cardiovascular event(s) during study period (Based in ICD-10-CA classifications)	Hospital Services
	Myocardial infarction (I21. - I23.)	
	Stroke (all causes) (I60. - I64.)	
	Transient ischemic attack (G45.0)	
	Unstable angina (I20.0)	
	Other ischemic heart disease (I24. - I25.)	
	Revascularization procedures	
	PTCA (1.IJ.50, 1.IJ.57.GQ, 1.IL.35), CABG (1.IJ.76)	
	Coronary Angiography (3.IP.10)	

Chronic disease score	Chronic disease score (measure of chronic disease status derived from population-based automated pharmacy data) [37]	Prescription Drug

Prior hospitalizations	Number of hospitalizations in one year prior to subject's index date	Hospital Services

Study hospitalizations	Number of hospitalizations during study observation period	Hospital Services

Physician visits	Number of physician visits during and prior to observation	Physician Services

***Pharmacy Level***		

Pharmacy Type	Indicates the type of pharmacy (mass merchandise, chain/grocery, independent) [38]	Investigators to categorize

Pharmacy Location	Rural (population < 10,000)	Investigators to categorize
	Regional (population 10,000 - 100,000)	
	Urban (population > 100,000)	

Prescription Volume	Mean number of new statin prescriptions filled monthly (proxy measure for prescription volume)	Prescription Drug

Pharmacist Coverage	Pharmacist overlap or single pharmacist	Pharmacy survey

Technician Support	Pharmacy technician support	Pharmacy survey

Two secondary endpoints, the proportion of new statin users who exhibit optimal adherence (≥80%) at one-year and mean persistence will be measured on an individual level and analyzed using GEE with an exchangeable correlation matrix and robust standard errors. All analyses will be intention-to-treat, and will include any pharmacies that have withdrawn, as well as any individual subjects who are no longer beneficiaries of the Saskatchewan Drug Plan (due to death or coverage termination). Mean imputation will be used for subjects or pharmacies lost-to-follow-up.

Ethics approval for the study protocol was obtained from the University of Saskatchewan Biomedical Ethics Board (09-135). The study is registered at ClinicalTrials.gov no. NCT00971412.

## Discussion

Medication non-adherence is a global problem [28] that consumes substantial financial health care resources [29], and has been recognized as a predictor of negative patient outcomes [17,30]. Non-adherence is complicated and multifactorial [31], and health interventions that are aimed at improving it are often not evaluated using pragmatic study designs. Recognizing this, we have utilized a novel study design evaluating an intervention involving community pharmacies, aimed at improving medication adherence.

A CRT design was selected for this study for two reasons. First, the intervention is intended for implementation at the community pharmacy level, so it is logical that community pharmacies would be the unit of randomization. Second, this design will lessen the risk for experimental contamination because pharmacists will not be forced to provide intervention and usual care activities at the same time [32]. We can not rule out the potential for the Hawthorne effect [33] within the usual care pharmacies; however, the effect is likely minimal as this group was intentionally not given any indication of the focus of the study intervention (adherence of new statin users). Also, this design will eliminate the need to recruit, obtain consent, and randomize individual patients, which is often impractical in typical community pharmacy settings.

We were prudent in following the guidelines for cluster randomized trials outlined in the CONSORT statement [34], ensuring that the risk of contamination between groups will be minimized, while maintaining the integrity of the experimental group comparison. Because the intervention will be evaluated in all new statin users presenting to each store, it will enhance the external validity of this study. Also, by ensuring all patient data are collected at arms length (by the Saskatchewan Ministry of Health), pharmacists can implement the adherence support strategy on all patients without the administrative burden of obtaining patient consent and collecting study specific information.

In addition, we have also provided the ICC used in estimating our sample size. To our knowledge, this ICC is the first one to be published in this particular field of research. We hope this value will prove useful in the design of future studies as well as encourage other researchers, especially in the area of medication adherence, to share their ICCs or cluster specific event rates.

In conclusion, we have presented, in detail, the design of the CPATCH study. Our approach combines the rigor of a randomized trial with a pragmatic approach to implementing and recording the results in a real-world fashion. We believe this approach can serve as an example of study design for the evaluation of future practice-based adherence interventions in large randomized trials.

## Competing interests

The authors declare that they have no competing interests.

## Authors' contributions

CDE conceived the study, participated in its design, and drafted the manuscript. DTE and JGT participated in the study design, and helped to draft the manuscript. AJR and YMS were responsible for critical review of the manuscript. DFB conceived the study, participated in its design and coordination, and helped to draft the manuscript. All authors read and approved the final manuscript.
